# The effectiveness and potential regulatory mechanism of *Blumea balsamifera* derived extracellular vesicles in promoting burn wound healing

**DOI:** 10.3389/fcell.2026.1756718

**Published:** 2026-03-19

**Authors:** Lei Chen, Linli Jiang, Xue Han, Rui Zhang, Fengping Zhao, Gengqi Fan, Yueping Li, Wei Han, Wenyuan Chen, Xiaolan Chen

**Affiliations:** 1 College of Pharmacy, Guizhou University of Traditional Chinese Medicine, Guiyang, Guizhou, China; 2 School of Pharmacy, Bijie Medical College, Bijie, Guizhou, China

**Keywords:** anti-inflammatory, Blumea balsamifera (L.) DC, burns, PI3K, AKT, mTOR signaling pathway, plant-derived extracellular vesicles

## Abstract

**Introduction:**

*B. balsamifera* is a medicinal plant traditionally used for burn treatment in Chinese folk medicine. Although *B. balsamifera* oil promotes wound healing, its clinical application is limited by volatility and skin irritation. Plant-derived extracellular vesicles, characterized by excellent biocompatibility, low irritancy, and ease of formulation, represent a promising alternative for wound therapy.

**Methods:**

In this study, a method for the extraction of *B. balsamifera*-derived extracellular vesicles (BB-DEVs) was successfully established, and their key physicochemical properties were characterized. Subsequently, the miRNA expression patterns in *B. balsamifera* leaves and BB-DEVs were analyzed using high-throughput sequencing. Additionally, network pharmacology was employed to predict the potential targets of BB-DEVs in the treatment of burns and scalds. Finally, a mouse model of scald injury on the dorsal skin was established to evaluate the therapeutic efficacy of BB-DEVs, and the underlying mechanisms were further explored using ELISA and Western blotting.

**Result:**

In this study, we isolated extracellular vesicles from *B. balsamifera* as a biocompatible nanoplatform and investigated their role and mechanism in burn healing. BB-DEVs were successfully isolated by differential centrifugation, exhibiting an appropriate size distribution and morphology. GC–MS analysis identified 95 components, including terpenes, terpenoids, fatty acids and derivatives, and aromatic compounds. miRNA sequencing of BB-DEVs and B. balsamifera leaves revealed differentially expressed miRNAs, whose potential cross-kingdom human gene targets were predicted. Network pharmacology analysis further intersected these targets with known burn-related genes. KEGG enrichment indicated significant involvement of the PI3K–Akt pathway (P < 0.01). *In vitro* and *in vivo* experiments confirmed the anti-inflammatory effect of BB-DEVs, which significantly reduced the levels of IL-6 and TNF-α and increased the level of IL-10. Western blot analysis confirmed elevated phosphorylation levels of PI3K, AKT, and mTOR proteins.

**Discussion:**

Our study reveals the potential of BB-DEVs in promoting burn wound healing.

## Graphical Abstract

**Figure:**
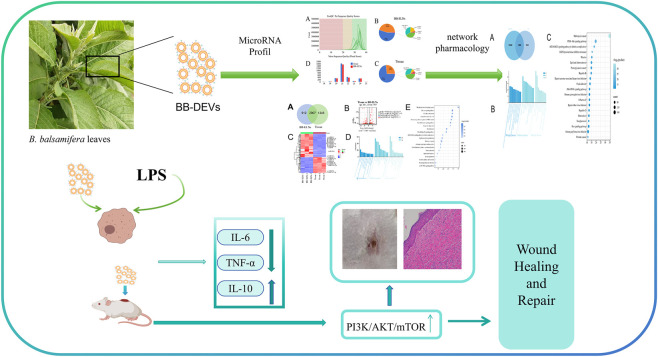
Graphical summary; Nanovesicles were confirmed to be present in B. balsamifera leaves. The results of high-throughput sequencing combined with network pharmacology showed that BB-DEVs treatment of burns may be related to the PI3K/AKT signaling pathway, and *in vivo* experiments confirmed this. It was also confirmed that BB-DEVs had anti-inflammatory ability.

## 1 Introduction

Burns and scalds are among the most common traumatic injuries worldwide, with a high incidence affecting approximately 8.3 million people annually (Yin et al., 2025). Severe burns can lead to damage of the musculoskeletal and nervous systems (Auger et al., 2017; Kankam et al., 2022; Duke et al., 2019)and are frequently accompanied by psychological sequelae, such as depression and anxiety (Giannoni-Pastor et al., 2016). Inflammation is a predominant complication following burn injury. In severe cases, this response can become excessive and dysregulated, significantly delaying wound healing (Jeschke et al., 2020). Pro-inflammatory and anti-inflammatory cytokines, including interleukin (IL)-6, IL-10, and tumor necrosis factor (TNF)-α, play pivotal roles in the repair process, and their expression levels serve as key indicators for evaluating therapeutic agents for scald injuries (Yu et al., 2023). Consequently, controlling the excessive inflammatory response represents a critical strategy for promoting the healing of burn wounds.

Exosomes, which are cell-secreted vesicles with diameters ranging from 30 to 150 nm (Kalluri and LeBleu, 2020), are widely present in both animals and plants (Zhao et al., 2024; Zhao et al., 2022). They carry various bioactive molecules, including miRNAs, mRNAs, DNAs, proteins, and lipids (Zhang et al., 2022) and play important regulatory roles in the pathophysiology of numerous human diseases (Zuo et al., 2019; Zumaquero et al., 2010; Zou et al., 2023a). Plant-derived extracellular vesicles (PDEVs) share a similar nanoscale vesicular structure with mammalian exosomes and likewise exhibit the capacity to modulate disease processes. For instance, nanovesicles derived from grapefruit have demonstrated potential as agents for wound healing (Savcı et al., 2021), and ginseng-derived nanoparticles can promote cutaneous wound repair via the ERK and AKT/mTOR pathways (Yang et al., 2023). Previous studies indicate that PDEVs often retain medicinal properties analogous to those of their source plants. A notable example is Aloe vera-derived nanovesicles, which possess anti-inflammatory activity, inhibit myofibroblast differentiation, and show promise in promoting wound healing (Ramírez et al., 2024). These effects are consistent with the known pharmacological properties of Aloe vera (Tazeze et al., 2021; Zahra et al., 2024; Razia et al., 2022). These findings collectively underscore the therapeutic potential of PDEVs in human health. However, a comprehensive understanding of PDEV biology, particularly at the transcriptomic level, remains limited. The scarcity of transcriptomic data on PDEVs hampers a clear elucidation of their biological functions (Fernandes et al., 2020). m MicroRNAs (miRNAs) are small, non-coding, single-stranded RNAs highly enriched within exosomes (Pegtel and Gould, 2019). They regulate target gene expression through base-pair complementarity and are extensively involved in critical cellular processes such as proliferation, differentiation, migration, and apoptosis, as well as in disease pathogenesis and progression (Hill and Tran, 2021; Lukasik and Zielenkiewicz, 2016). One potential key mechanism underlying PDEV bioactivity involves cross-kingdom regulation of host genes via delivered plant miRNAs. Although this is still an active topic of debate. Therefore, profiling the miRNA of PDEVs is essential for elucidating their pharmacological mechanisms.

*Blumea balsamifera* (L.) DC., a perennial plant of the Compositae family, exhibits diverse pharmacological properties, including antimicrobial, anti-inflammatory, analgesic, antitumor, and wound-healing activities (Pang et al., 2017; Peng et al., 2025; Jirakitticharoen et al., 2022). The essential oil extracted from *B. balsamifera* (*B. balsamifera* oil, BBO) possesses additional benefits such as antimicrobial (Yang et al., 2021) and antioxidant effects (Yuan et al., 2016). Traditionally used in ethnic medicine, the wound-healing efficacy of B. balsamifera is well-documented. For instance, BBO has been shown to promote burn wound repair and reduce scar formation (Fan et al., 2015), alleviate UV radiation-induced skin erythema and epidermal thickening in mice (Zhang et al., 2021), and its flavonoid constituents enhance skin wound healing in rats (Pang et al., 2017). These findings underscore the significant therapeutic potential of *B. balsamifera* in wound management. Concurrently, plant-derived extracellular vesicles (PDEVs) have emerged as promising candidates for skin wound therapy owing to their superior biocompatibility, low irritancy, and favorable formulation properties (Zhang et al., 2016). Building on this, the present study aimed to investigate the role of *B. balsamifera*–derived extracellular vesicles (BB-DEVs) in burn wound healing. We characterized the morphology and particle size of BB-DEVs and profiled their miRNA content via small RNA sequencing. *In vitro* and *in vivo* experiments demonstrated that BB-DEVs modulate the levels of key inflammatory cytokines (IL-6, TNF-α, and IL-10) and significantly promote burn wound repair. Collectively, our results reveal the therapeutic potential of BB-DEVs for burn wound healing.

## 2 Materials and methods

### 2.1 Isolating and purification of BB-DEVs

Fresh leaves of *B. balsamifera* were rinsed with water, soaked in phosphate-buffered saline (PBS) for 12 h, and homogenized using a high-speed blender for 8 min. The resulting homogenate was sequentially centrifuged at 3,000 × g for 30 min and 5,000 × g for 50 min (H2050R, Cence, China) to remove large cellular debris. The supernatant was collected and further centrifuged at 15,000 × g for 60 min to obtain a pellet. This pellet was resuspended in PBS and subjected to sucrose density gradient centrifugation (prepared with 8%, 30%, 45%, and 60% (w/v) sucrose layers) at 16,680 × g for 90 min. The band observed at the interface between the 8% and 30% sucrose layers was carefully collected and centrifuged again at 16,680 × g for 90 min at 4 °C. Finally, the supernatant was discarded, and the pellet was resuspended, passed through a 0.22 μm filter, and stored at −80 °C. All freshly isolated BB-DEVs were immediately aliquoted and stored at −80 °C to minimize freeze-thaw cycles and long-term degradation. For subsequent experiments, BB-DEVs were thawed only once and used within 2 h after thawing.

### 2.2 Physicochemical characterization

The BB-DEVs were adsorbed onto a carbon-coated copper grid and negatively stained with 2% phosphotungstic acid for 1–2 min. Morphological observation was performed using a transmission electron microscope (JEM-1400FLASH, JEOL, Tokyo, Japan). The particle size distribution was analyzed with a Zetasizer Lab system (Malvern Instruments Ltd., Malvern, United Kingdom). Particle concentration and distribution were measured using a nanoparticle tracking analyzer (ZetaView, Particle Metrix).

### 2.3 Protein quantification

The protein concentration of the BB-DEV samples was determined using a bicinchoninic acid (BCA) assay kit (Solarbio, China) according to the manufacturer’s instructions.

### 2.4 Composition analysis of BB-DEVs

The chemical composition of BB-DEVs was analyzed by gas chromatography–mass spectrometry (GC-MS; Agilent, United States). BB-DEVs was placed into a 20 mL headspace vial, mixed with saturated sodium chloride solution, and spiked with the internal standard 2-octanol at a final concentration of 3 mg/L. The vial was then heated at 80 °C for 30 min. Subsequently, a the headspace micro-extraction injection needle was inserted into the vial, and the assembly was heated for an additional 30 min, followed by thermal desorption at 250 °C for 5 min. Chromatographic conditions were as follows: column, HP-5MS (30 m × 0.25 mm × 0.25 μm); temperature program: held at 50 °C for 2 min, increased to 180 °C at 5 °C/min and held for 5 min, then raised to 250 °C at 10 °C/min and held for 5 min; injector temperature, 250 °C; transfer line temperature, 280 °C; carrier gas flow rate, 1.0 mL/min; split ratio, splitless. Mass spectrometry conditions were as follows: ion source, electron impact (EI); ion source temperature, 230 °C; quadrupole temperature, 150 °C for 5 min. Chromatographic conditions: chromatographic column: HP-5MS (30 m × 0.25 mm × 0.25 μm) ; column temperature: the initial temperature was maintained at 50 °C for 2 min, increased to 180 °C at 5 °C/min for 5 min, and then increased to 250 °C at 10 °C/min for 5 min. Injector temperature: 250 °C; transmission line temperature: 280 °C; carrier gas flow rate: 1.0 mL/min; shunt ratio: no shunt. Mass spectrometry conditions: ion source temperature: 230 °C, quadrupole temperature: 150 °C, mass spectrometry: EI source.

### 2.5 Total RNA preparation

Total RNA was isolated from both BB-DEVs and source plant tissue for small RNA sequencing, using the Qiagen miRNeasy Serum/Plasma Kit according to the manufacturer’s protocol. Briefly, five volumes of QIAzol Lysis Reagent were added to each sample and mixed thoroughly, followed by incubation at room temperature (15 °C–25 °C) for 5 min. An equal volume of chloroform was added, the mixture was vortexed vigorously for 15 s, and then incubated at room temperature for 3 min. After centrifugation at 12,000 × g for 15 min at 4 °C, the aqueous phase was transferred to a new tube and mixed with 1.5 volumes of 100% ethanol. A volume of 700 μL of the mixture was loaded onto a RNeasy MinElute spin column and centrifuged at 8,000 × g for 15 s at room temperature; the flow-through was discarded. The column was washed sequentially with 700 μL of Buffer RWT (centrifuged at 8,000 × g for 15 s) and twice with 500 μL of Buffer RPE (centrifuged at 8,000 × g for 15 s), discarding the flow-through after each step. Subsequently, 500 μL of 80% ethanol was added, and the column was centrifuged at 8,000 × g for 2 min. The column was then transferred to a fresh 2 mL collection tube and centrifuged at 12,000 × g for 1 min. Finally, the column was placed in a clean 1.5 mL collection tube, and 30 μL of RNase-free water was applied directly to the center of the membrane. After a 2-min incubation at room temperature, the column was centrifuged at 8,000 × g for 1 min to elute the RNA. The eluted RNA was collected and stored at −80 °C.

### 2.6 RNA Qsep100 assay

RNA concentration was determined using a fluorometric nucleic acid analyzer (Quantus Fluorometer, Promega). Based on the measured concentration, each RNA sample was diluted with the appropriate cartridge-specific diluent and analyzed for integrity by automated capillary electrophoresis (Qsep100, BIOptic).

### 2.7 Small RNA sequencing for BB-DEVs

Small RNA sequencing libraries were prepared using the PE150 paired-end protocol. Sequencing quality was assessed with FastQC. Raw reads were then processed with fastp to trim adapter sequences, remove terminal N-bases, and filter out reads with quality scores below Q20. The cleaned reads were aligned against the Rfam database using Bowtie to remove ribosomal RNA (rRNA), transfer RNA (tRNA), and other non-coding RNAs (ncRNAs). miRNA expression was quantified using miRDeep2, and differential expression analysis was performed with DESeq2.

### 2.8 Differential expression of miRNA

Differential expression analysis between BB-DEVs and source tissue was performed using DESeq2, with the tissue sample designated as the reference group. The resulting P-values were adjusted using the qvalue method. Differentially expressed miRNAs were defined as those with an adjusted P-value <0.05 and an absolute log2 fold change >1.

### 2.9 miRNA target gene prediction

For target gene prediction, the human genome was used as the reference. The top 10 upregulated and top 10 downregulated miRNAs from the differential expression analysis were selected to predict their potential targets on human genes using the miRanda algorithm. Functional enrichment analysis of the predicted target genes was subsequently performed based on the Gene Ontology (GO) and Kyoto Encyclopedia of Genes and Genomes (KEGG) databases.

### 2.10 Burn target gene acquisition

To identify burn-related genes, the keyword “burns” was queried in the GeneCards database (https://www.genecards.org/) (Mo et al., 2022) and all resulting gene entries were retrieved for subsequent analysis. The overlapping target genes between BB-DEVs and burn-related genes were then identified using the Venny online tool (https://www.bioinformatics.com.cn/).

### 2.11 GO and KEGG pathway analyses

The overlapping target genes obtained from Section 2.9 were submitted to the DAVID database (https://david.ncifcrf.gov/) for GO annotation and KEGG pathway enrichment analysis, in order to investigate the principal biological functions and key signaling pathways associated with BB-DEVs in burn treatment. The analysis was performed with the species set to *Homo sapiens* and a significance threshold of *p* < 0.05 (Cui et al., 2022). Results were visualized as a bar chart for GO enrichment and a bubble chart for KEGG enrichment.

### 2.12 Anti-inflammatory effects of BB-DEVs

RAW264.7 cells were cultured in DMEM supplemented with 10% fetal bovine serum (FBS) and 1% penicillin–streptomycin at 37 °C in a humidified atmosphere containing 5% CO_2_.

To evaluate the anti-inflammatory potential of BB-DEVs, an LPS-stimulated RAW264.7 cell model was used, and the secretion of inflammatory factors was analyzed. Cells were seeded into 96-well plates at a density of 1 × 10^4^ cells per well and cultured overnight to allow attachment and stabilization. The culture medium was then replaced with medium containing BB-DEVs at various concentrations (10, 20, 40, 80, 160, 320, and 640 μg/mL, quantified based on protein content) for 24 h. After treatment, the supernatant was removed, and cell viability was assessed using the CCK-8 assay according to the manufacturer’s instructions. Briefly, CCK-8 working solution was added to each well and incubated for 2 h, after which the absorbance at 450 nm was measured. Cell viability was calculated using the following formula:

$$CV\left(\%\right)=\frac{A_{m}-A_{n}}{A_{k}-A_{n}}\times 100\%$$

Where A_m_ represents the absorbance of BB-DEVs group, A_n_ represents the absorbance of blank group, and A_k_ represents the absorbance of control group.

RAW264.7 cells were seeded in 96-well plates at a density of 1 × 10^4^ cells/mL and cultured overnight at 37 °C under 5% CO_2_ to allow cell adhesion. The medium was then replaced with fresh medium containing 1 μg/mL lipopolysaccharide (LPS) and various concentrations of BB-DEVs (10, 20, and 40 μg/mL). After 24 h of incubation at 37 °C and 5% CO_2_, the culture supernatant was collected and centrifuged at 1,500 rpm for 15 min at 4 °C. Levels of IL-6, IL-10, and TNF-α in the supernatant were measured using commercial ELISA kits according to the manufacturer’s protocols.

### 2.13 Experimental animals

Male KM mice (8 weeks old) were purchased from SCBS Biotechnology Co., Ltd. (Henan, China). All animals were housed under specific pathogen-free conditions with *ad libitum* access to food and water. Animal care and experimental procedures followed the institutional guidelines of the Guizhou University of Traditional Chinese Medicine and were approved by its Laboratory Animal Welfare Ethics Committee (Animal Experiment Ethics No. 20250331002). The study was conducted under the project license XYXK (Qian) 2021–0005.

### 2.14 Establishment of the scald/burn model and treatment of mice

Mice were randomly assigned to six groups (n = 6 per group): control (sham-treated), model (burn injury without treatment), positive drug (treated with “Jing wan hong” ointment), BB-DEVs low-dose, BB-DEVs high-dose, and BBO (treated with *B. balsamifera* essential oil). All mice were anesthetized by intraperitoneal injection of 10% urethane (1 g/kg). The dorsal fur was shaved, and residual hair was removed with depilatory cream. A second-degree scald injury was induced using a temperature-controlled scald instrument (YLS-5Q, Jinan Yiyan Technology Development Co., Ltd., China) set at 100 °C. A uniform heating probe was placed firmly on the depilated dorsal skin for 5 s. Starting 24 h post-injury, wounds were treated once daily for 21 consecutive days according to group assignment: control group received 2 mL of 0.01 M PBS; positive group received 0.1 g of “Jing wan hong” ointment; low-dose group received 100 μg of BB-DEVs suspension; high-dose group received 200 μg of BB-DEVs suspension; model group received no treatment; and the BBO group received topical application of *B. balsamifera* essential oil.

### 2.15 Measurement of wound healing rate

All treatments were applied daily at the same fixed time. Wound images were captured on days 1, 5, 9, 13, 17, and 21 post-modeling. The wound area in each image was measured using ImageJ software, and the wound contraction percentage for a given day was calculated as:

$$\text{Wound contraction }\left(\%\right)=\left(\text{Wound area on day }X\;/\text{ Initial wound area}\right)\times 100.$$

### 2.16 Histopathological examination

On day 21 post-modeling, mice in each group were anesthetized by intraperitoneal injection of 10% urethane (1 g/kg). Approximately 1.5 mL of blood was collected from the orbital sinus, allowed to clot for 2 h at room temperature, and then centrifuged at 3,000 rpm for 20 min. The upper serum layer was carefully transferred to a clean tube and stored at −80 °C for subsequent analysis.Wound tissue samples were fixed in 4% paraformaldehyde, embedded in paraffin, and sectioned. Sections were stained with hematoxylin and eosin (H&E) for histological examination.

### 2.17 ELISA

Frozen serum samples were thawed and analyzed for the concentrations of tumor necrosis factor-α (TNF-α), interleukin-6 (IL-6), and interleukin-10 (IL-10) using commercial enzyme-linked immunosorbent assay (ELISA) kits (ZCIBIO Technology Co., China) according to the manufacturer’s instructions.

### 2.18 Western blot

Wound skin tissues were weighed and homogenized in RIPA lysis buffer (tissue weight: buffer volume = 1: 10). After centrifugation, the supernatant was collected, and total protein concentration was determined using a BCA protein assay kit. Proteins were separated by SDS-PAGE and transferred onto PVDF membranes. The membranes were blocked with 5% non-fat dry milk for 1 h at room temperature, followed by overnight incubation at 4 °C with the following primary antibodies: phospho-PI3K, phospho-AKT, and phospho-mTOR (Servicebio, China). After washing five times with TBST (5 min per wash), membranes were incubated with appropriate horseradish peroxidase-conjugated secondary antibodies for 1 h at 37 °C. Protein bands were visualized using an ECL chemiluminescence substrate. Band intensities were quantified with ImageJ software, and relative protein expression levels were normalized to GAPDH as the loading control.

### 2.19 Statistical analysis

Statistical analysis was performed with SPSS 24.0 software. One-Way ANOVA test is used for comparison among multiple groups. All data were from at least 3 independent experiments. *p* < 0.05 were considered statistically significant.

## 3 Results

### 3.1 Characterization of BB-DEVs

**FIGURE 1:**
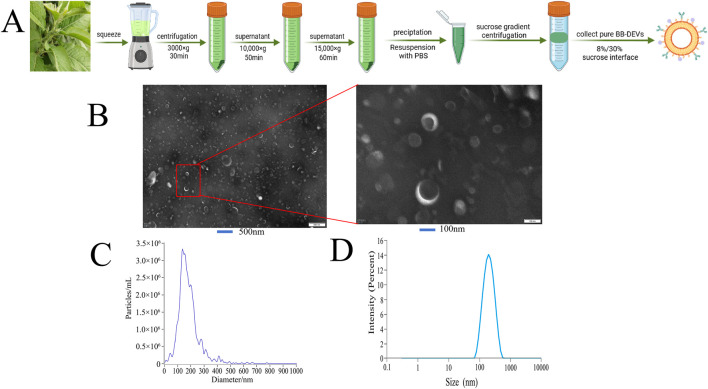
**(A)** Extraction of BB-DEVs from *B. balsamifera* leaves; **(B)** TEM characterization of BB-DEVs; **(C)** The number of BB-DEVs was analyzed by NTA; **(D)** The particle size of BB-DEVs was analyzed by particle size analyzer.

BB-DEVs were isolated from *B. balsamifera* leaf juice and purified through differential centrifugation followed by sucrose density gradient ultracentrifugation (Figure 1A). The vesicles accumulated predominantly at the 8%/30% sucrose interface. BB-DEVs were characterized for morphology, size, particle concentration, and protein content. As shown in Figure 1B, BB-DEVs displayed a typical rounded vesicular morphology consistent with extracellular vesicles. Nanoparticle tracking analysis (NTA) revealed a total particle concentration of 2.1 × 10^11^ particles/mL (Figure 1C) with an average particle size of 173.3 ± 0.85 nm (Figure 1D). The zeta potential was measured at −18.57 ± 0.39 mV, and the protein concentration of the BB-DEV preparation was 252.9 μg/mL.

### 3.2 Component analysis of BB-DEVs

GC-MS analysis revealed 95 major constituents in BB-DEVs (Supplementary Table S1), including terpenes and terpenoids, fatty acids and their derivatives, aromatic compounds, and oxygen-/nitrogen-containing aromatic and heterocyclic compounds. Notably, BB-DEVs contained components also present in *B*. *balsamifera* leaves, such as linalool and caryophyllene oxide (Yuan et al., 2016). These compounds have been reported to possess anti-inflammatory and analgesic properties (Jiang et al., 2015; Lal et al., 2025). These results suggest that BB-ELNs may be a nanovesicles with therapeutic potential.

### 3.3 Small RNA sequencing result

**FIGURE 2:**
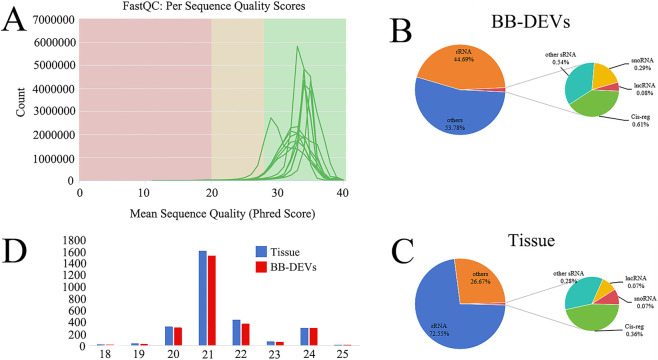
**(A)** Distribution of sequencing quality; **(B)** Statistics of sRNA classification of BB-DEVs; **(C)** Statistics of sRNA classification of *B. balsamifera* leaves.; **(D)** Length distribution of clean reads of BB-DEVs and *B. balsamifera* leaves small RNA libraries.

Small RNA sequencing was performed on isolated BB-DEVs. To identify miRNAs specifically enriched in BB-DEVs, RNA from B. balsamifera leaf tissue was analyzed in parallel as a reference. Six small RNA libraries were constructed (two sample types with three biological replicates each). During sequencing, base-calling accuracy was monitored using the Phred quality score, and the Q30 threshold was met for all libraries (Figure 2A). Annotation of small RNAs revealed distinct distributions of various small RNA classes between BB-DEVs and leaf tissue (Figures 2B,C). In both BB-DEVs and tissue, the majority of miRNAs fell within the 20–24 nt length range (Figure 2D).

### 3.4 Differential expression analysis of miRNA between tissue and BB-DEVs

**FIGURE 3:**
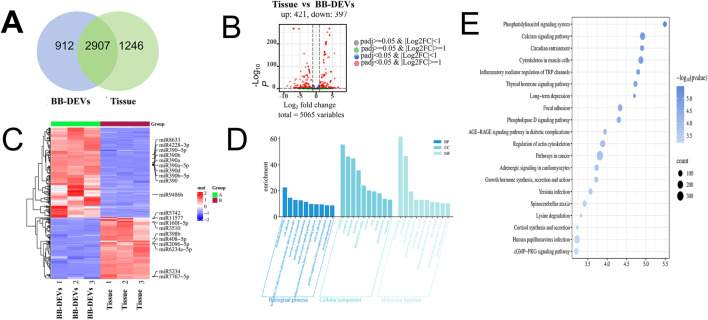
**(A)** Venn diagram of the intersection of miRNAs in BB-DEVs and *B. balsamifera* leaves; **(B)** Volcano plot of the different expression patterns between *B. balsamifera* leaves and BB-DEVs. Note: The red solid circle satisfies both padj <0.05 and |Log2FC| ≥ 1 (FC: multiples of difference between the two samples), with significant differences. Log2 (FC) < 0, downregulated miRNA (Tissue VS BB-DEVs); Log2 (FC) > 0, upregulated miRNA (Tissue VS BB-DEVs). **(C)** Heatmap of differential miRNAs between BB-DEVs and Tissue. The heatmap is based on the TPM. **(D)** GO functional enrichment of Top 20 miRNA target genes. **(E)** KEGG functional enrichment of Top 20 miRNA target genes.

A total of 5,056 miRNAs were identified across BB-DEVs and tissue samples. Of these, 2,907 miRNAs were commonly expressed in both sample types, whereas 912 miRNAs were unique to BB-DEVs and 1,246 were unique to tissue (Figure 3A). A total of 818 miRNAs were differentially expressed between BB-DEVs and tissue, comprising 421 upregulated and 397 downregulated miRNAs (Figure 3B,C; Supplementary Table S2).

### 3.5 Target genes prediction of the top 10 up- and downregulated miRNAs

This study focused on the top 10 upregulated and top 10 downregulated miRNAs enriched in BB-DEVs. These miRNAs were analyzed using the miRanda algorithm to predict their human target genes, yielding a total of 11,722 predicted targets (Supplementary Table S3). GO and KEGG annotations were subsequently performed. GO analysis indicated that the predicted target genes were associated with diverse functions: under biological processes, they were primarily linked to cellular signaling, growth regulation, and inflammatory response; in cellular components, they were enriched for cell structures, membrane systems, organelles, and neuron-specific structures; and within molecular functions, they were mainly involved in receptor activity, protein-interaction motifs, and kinase-related activities (Figure 3D; Supplementary Table S4). KEGG pathway analysis (Figure 3E; Supplementary Table S5) revealed that the top 20 enriched pathways included Phosphatidylinositol signaling system, Inflammatory mediator regulation of TRP channels, and Calcium signaling pathway.

### 3.6 Potential targets of compound therapy for burn

**FIGURE 4:**
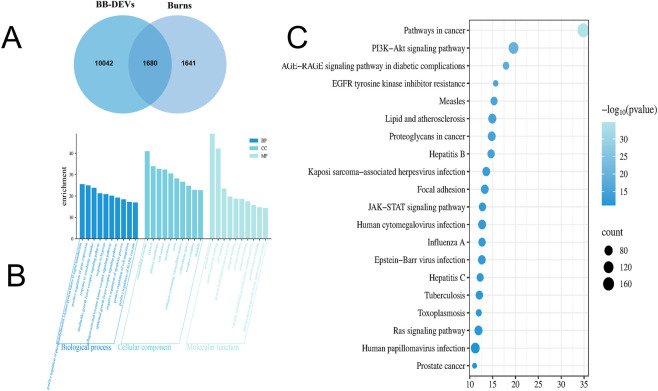
**(A)** Venn diagram of BB-DEVs target genes and burn target genes. **(B)** GO enrichment analysis of targets of BB-DEVs in treating burn. **(C)** KEGG pathway enrichment analysis of targets of BB-DEVs in treating burn.

A query of the GeneCards database yielded 3,321 burn-associated targets (Supplementary Table S6). A Venn diagram generated between these burn-related targets and the predicted targets of BB-DEVs revealed 1,680 overlapping genes (Figure 4A; Supplementary Table S7).

### 3.7 GO and KEGG pathway enrichment analysis

GO and KEGG enrichment analyses of the overlapping targets between BB-DEVs and burn-related genes were performed using the DAVID database, with the species restricted to *Homo sapiens*. A total of 612 GO terms were significantly enriched (FDR <0.01), comprising 364 biological process (BP), 139 cellular component (CC), and 109 molecular function (MF) terms. The top 10 terms in each category are displayed in a bar chart (Figure 4B; Supplementary Table S8). Among BP terms, the most enriched included positive regulation of phosphatidylinositol 3-kinase/protein kinase B signaling, positive regulation of gene expression, and response to xenobiotic stimulus. In the CC category, targets were primarily localized to the extracellular exosome, cytosol, and plasma membrane. For MF, the main activities involved protein binding, identical protein binding, and ATP binding. KEGG pathway enrichment indicated that BB-DEVs may significantly affect pathways in cancer, the PI3K-Akt signaling pathway, and the AGE-RAGE signaling pathway in diabetic complications. The top 20 enriched pathways are presented as a bubble plot (Figure 4C; Supplementary Table S9).

### 3.8 BB-DEVs affect the secretion of cytokines

**FIGURE 5:**
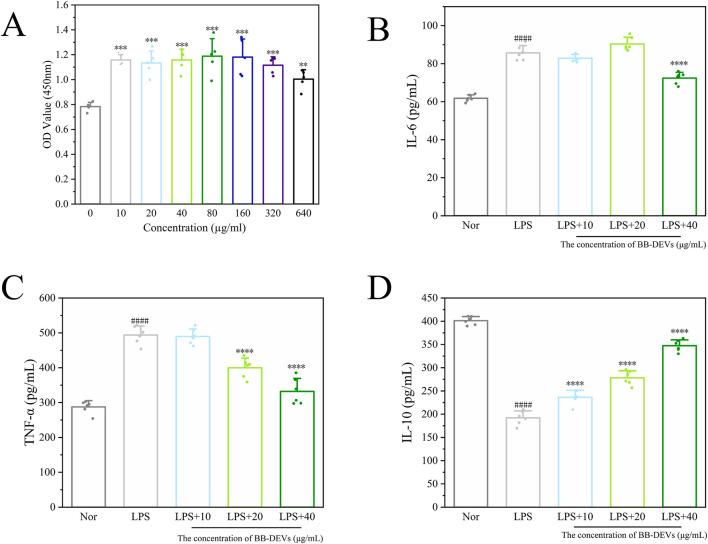
**(A)** Survival of RAW264.7 cells after different treatments. (B/C/D) The effects of different treatment groups on the secretion of IL-10 **(B)**, TNF-α **(C)** and IL-6 **(D)** in RAW264.7 cells induced by LPS. (Mean ± SEM, n = 6, #*p* < 0.05, ##*p* < 0.01, ###*p* < 0.001, ####*p* < 0.0001compared with the normal group; *p < 0.05, **p < 0.01, ***P < 0.001, ****P < 0.0001comparison with model group).

To assess the anti-infammatory potential of BB-DEVs, RAW264.7 cells were stimulated with LPS in the presence of varying concentrations of BB-DEVs. Within the tested concentration range, BB-DEVs exhibited no cytotoxicity toward RAW264.7 cells (Figure 5A). LPS stimulation signifcantly elevated the secretion of IL-6 and T’NF-a while suppressing IL-10 release. Treatment with 40 μg/mL, BB-DEVs markedly reduced the levels of IL-6 (Figure 5B) and ‘TNF-a (Figure 5C), and increased the level of IL-10 (Figure 5D).

### 3.9 BB-DEVs promote second-degree burn wound healing

**FIGURE 6:**
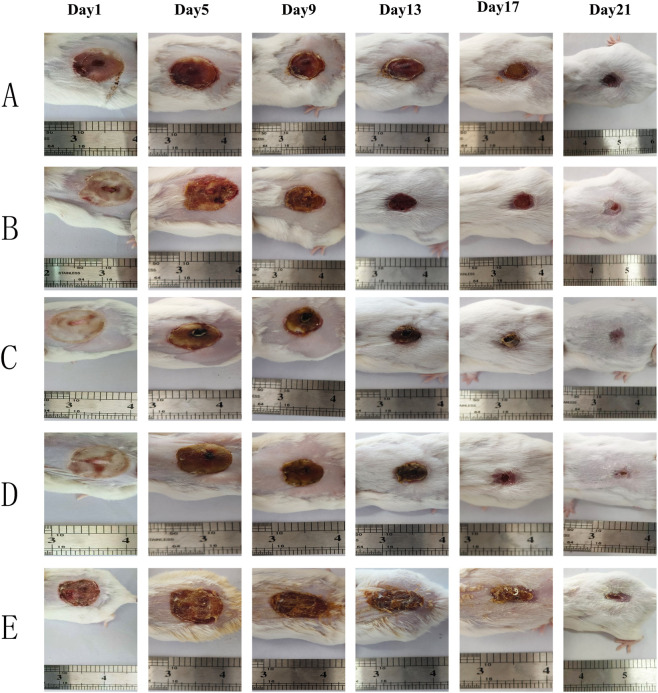
Photographs of burn wound repair process on the back of mice. Note: **(A)** model group; **(B)** positive drug group; **(C)** low dose group; **(D)** high dose group; **(E)** BBO group.

We evaluated the potential of BB-DEVs to promote burn wound healing through *in vivo* experiments. Wound photographs from each group were scaled uniformly to visualize healing progress (Figure 6). On day 5, wound areas showed no marked change, and hard scabs were present in all groups. By day 9, wound areas had decreased noticeably in all groups, though inter-group differences remained insignificant. On day 13, scabs had detached and wound areas further reduced; compared with the model group, the positive-control and high-dose groups exhibited significantly smaller wounds. By day 21, wounds in all groups showed improvement, with those in the positive-control and high-dose groups nearly healed and lacking obvious scarring. The healing rates in the positive-control and high-dose groups were 96.20% and 95.66%, respectively, both significantly higher than those in the model and low-dose groups. The BBO group and low-dose group showed healing rates of 92.82% and 89.65%, respectively, also significantly greater than that of the model group (74.76%).

### 3.10 Histological analysis of regenerated skin tissue

**FIGURE 7:**
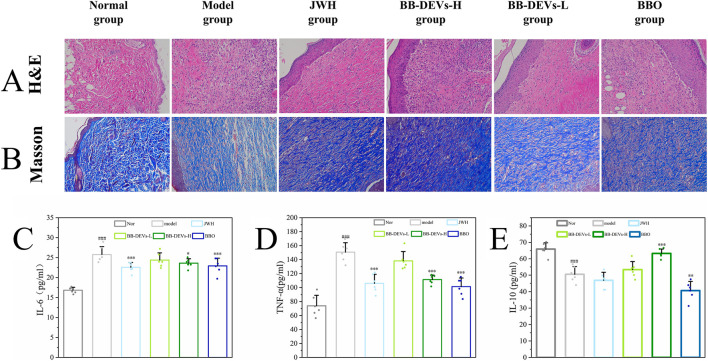
**(A,B)** H&E and Masson’s staining images of the wound. **(C–E)**: The anti-inflammatory effect of BB-DEVs. The levels of IL-6 **(C)**, TNF-α **(A)**, IL-10 **(E)** as determined by ELISA kits. (Mean ± SEM, n = 6, #*p* < 0.05, ##*p* < 0.01, ###*p* < 0.001 compared with the normal group; *p < 0.05, **p < 0.01, ***P < 0.001 comparison with model group).

Histopathological changes, collagen deposition, and inflammatory cell infiltration in mouse skin were assessed by H&E and Masson’s staining. H&E staining revealed an increased number of fibroblasts and capillaries in the model group; however, mild inflammatory cell infiltration and residual erythrocytes were still present, indicating persistent hemorrhage and incomplete wound healing by day 21. Compared with the model group, the treated groups showed greater increases in fibroblasts and capillaries, along with substantial formation of nascent sweat glands and no obvious erythrocytes, suggesting improved wound healing (Figure 7A).

Masson’s staining further illustrated the extent of collagen deposition. All treatment groups exhibited more collagen deposition than the model group. The “JWH” (positive control) and BB-DEVs-H (high-dose) groups displayed greater collagen deposition than the BB-DEVs-L (low-dose) and BBO groups (Figure 7B). These findings suggest that BB-DEVs promote collagen deposition in burn wounds, thereby facilitating wound healing.

### 3.11 Enzyme-linked immunosorbent assay (ELISA) results

The anti-inflammatory effect of BB-DEVs was further evaluated in a murine burn model by measuring serum levels of IL-6, IL-10, and TNF-α (Figures 7C–E). Compared with the normal group, the model group showed significantly elevated IL-6 and TNF-α levels and a marked reduction in IL-10. Relative to the model group, the JWH (positive control), BB-DEVs-H (high-dose), and BBO groups exhibited significant decreases in IL-6 and TNF-α, whereas the BB-DEVs-L (low-dose) group showed no significant change. Furthermore, the BB-DEVs-H group displayed a significant increase in IL-10 compared with the model group. One of the potential mechanisms through which BB-DEVs promote burn wound healing is mediated by their anti-inflammatory activity.

### 3.12 BB-DEVs treatment upregulates phosphorylation of PI3K, AKT, and mTOR in mice

**FIGURE 8:**
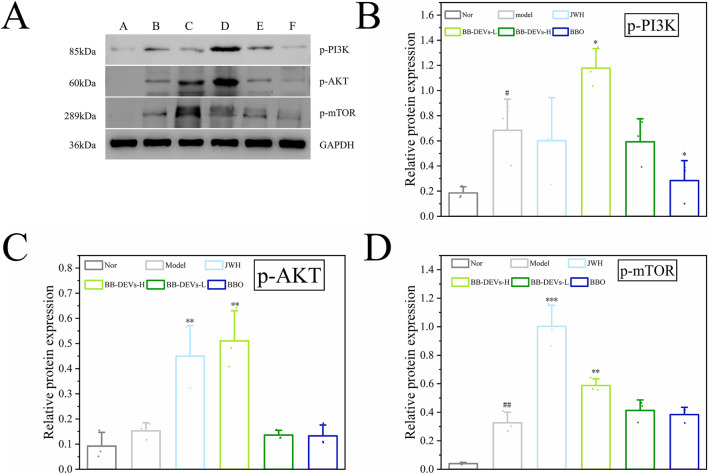
**(A)** Protein expression of p-PI3K, p-AKT, p-mTOR detected by Western blotting assay. A normal group; B model group; C positive drug group; D low dose group; E high dose group; F BBO group. **(B)**, **(C)** and **(D)** Optical density analysis of the relative expression levels of p-PI3K, p-AKT, p-mTOR (Mean ± SEM, n = 3, #*p* < 0.05, ##*p* < 0.01 compared with the normal group; *p < 0.05, **p < 0.01, ***p < 0.001comparison with model group).

KEGG enrichment analysis revealed predominant enrichment of overlapping genes in the PI3K-AKT pathway, whose activation plays a pivotal role in promoting wound healing (Gan et al., 2021). We therefore hypothesized that BB-DEVs might facilitate burn wound repair in mice by modulating the PI3K-AKT pathway. To investigate the underlying mechanism, we measured the phosphorylation levels of key proteins in this pathway. As shown in Figure 8, compared with the model group, the BB-DEVs-H group exhibited increased phosphorylation of PI3K, AKT, and mTOR. These results suggest that activation of the PI3K-AKT pathway may represent a potential mechanism through which BB-DEVs promote burn wound healing.

## 4 Discussion

Burn management remains a critical medical challenge. Burns can result from various sources, including thermal, electrical, frictional, radiative, and chemical agents. Beyond causing substantial physical damage, burn injuries also profoundly affect patients’ psychological wellbeing (Tolles, 2018; Richards et al., 2025; Żwierełło et al., 2023). underscoring the need for effective therapeutic strategies. In the present study, BB-DEVs were successfully isolated and shown to significantly accelerate burn wound healing in mice. These findings position BB-DEVs as promising therapeutic candidates for the treatment of burns.

Recent studies have increasingly identified PDEVs in diverse plant species and highlighted their potential benefits for human health (Zhang et al., 2016). Current methods for PDEV isolation include ultracentrifugation (Takakura et al., 2022), PEG precipitation (Liu et al., 2023), and size exclusion chromatography (Del Pozo-Acebo et al., 2022), among others. In this study, BB-DEVs were isolated via differential centrifugation coupled with sucrose density gradient purification, yielding nanovesicles with an average particle size of 173.3 nm. Transmission electron microscopy revealed that BB-DEVs exhibited a rounded or spherical morphology with a distinct lipid bilayer structure (Ramírez et al., 2024). GC-MS analysis demonstrated that BB-DEVs contained constituents also present in *B. balsamifera* leaves, such as linalool and caryophyllene oxide, along with various fatty acids and their derivatives. Subsequent measurement of vesicular protein concentration and small RNA sequencing confirmed that the isolated vesicles contained both protein and RNA components. These results suggest that BB-DEVs may possess anti-inflammatory properties similar to those of *B. balsamifera* and other reported PDEVs. Collectively, our results indicate that the described isolation method successfully obtained BB-DEVs and, owing to its relatively low equipment requirements, could be suitable for larger-scale production.

MiRNAs are recognized as key regulators in a broad spectrum of physiological processes, including cell proliferation, apoptosis, developmental timing, and immune responses (Zhao et al., 2018). PDEVs miRNAs possess the potential for cross-kingdom regulation. For example, lavender-derived extracellular vesicles carrying cpa-miR166e can effectively suppress the elevation of inflammatory factors (Li et al., 2025a). Exosome-like nanoparticles from Polygoni Multiflori Radix are taken up by human hair follicle dermal papilla cells, and their cargo miRNAs (aof-miR168a and osa-miR164a) significantly inhibit androgen receptor protein expression while increasing GSK3β phosphorylation (Li et al., 2025b). Predicting miRNA target genes and analyzing their functions can provide a theoretical foundation for the development and biomedical application of exosomes. In this study, we detected a total of 5,065 miRNAs in B. balsamifera leaves and BB-DEVs, of which 912 were exclusively detected in BB-DEVs and 1,246 were unique to the leaves. Comparative analysis revealed 818 differentially expressed miRNAs between BB-DEVs and leaf tissue. The top 20 most significantly differentially expressed miRNAs were selected for cross-kingdom human target prediction, yielding 8,732 potential target genes suitable for further exploration. These predicted targets were then intersected with known burn-related genes, and the overlapping gene set was subjected to GO and KEGG enrichment analyses. The results indicated that bioactive molecules carried by BB-DEVs are likely involved in functions related to intercellular connections and are significantly enriched in the PI3K-Akt signaling pathway. Activation of the PI3K/AKT/mTOR signaling pathway may play an important role in promoting wound healing (Xiong et al., 2023).

Evidence suggests that plant-derived extracellular vesicles (PDEVs) often retain medicinal properties analogous to their source plants. For example, both Aloe vera-derived extracellular vesicles and Aloe vera itself promote skin wound healing (Ramírez et al., 2024; Iosageanu et al., 2024). Similarly, ginseng-derived extracellular vesicles and the ginsenoside Rg5 each accelerate wound repair (Yang et al., 2023; Xia et al., 2023). In the present study, we demonstrated that BB-DEVs facilitate burn wound healing in mice. Persistent inflammation is a hallmark of burn wounds, and mitigating the inflammatory response effectively promotes healing (Zhao et al., 2025). Representative cytokines in inflammation include TNF-α, IL-10, and IL-6 (Zou et al., 2024; Zou et al., 2023b). Anti-inflammatory intervention is therefore crucial for burn wound recovery. In our *in vitro* and *in vivo* experiments, BB-DEVs significantly reduced the levels of IL-6 and TNF-α while elevating IL-10, indicating their anti-inflammatory potential. Western blot analysis further revealed that treatment with BB-DEVs upregulated the expression of phosphorylated PI3K, AKT, and mTOR, suggesting that BB-DEVs may activate the PI3K/AKT/mTOR signaling pathway. These findings suggest that both anti-inflammatory activity and activation of the PI3K/Akt/mTOR signaling pathway represent potential mechanisms through which BB-DEVs exert their therapeutic effects.

Although this study demonstrates the promising therapeutic potential of BB-DEVs in promoting burn wound healing, several limitations should be acknowledged. First, while we profiled the miRNA content of BB-DEVs and predicted their cross-kingdom targets, future work is needed to functionally validate the ability of these miRNAs to modulate gene expression across species. Second, although we identified anti-inflammatory constituents, miRNAs, and a nanovesicular structure in BB-DEVs, and confirmed their pro-healing and anti-inflammatory effects, a deeper understanding of their mode of action requires further dissection of the biological contributions of individual components. Subsequent studies should include gain- and loss-of-function experiments on specific miRNAs to delineate their precise roles in the observed therapeutic outcomes. Correspondingly, it will be crucial for future research to elucidate the individual and synergistic roles of the vesicular structure itself, alongside lipids, proteins, metabolites, and nucleic acids such as miRNAs.

In summary, this study characterized the composition and miRNA profile of BB-DEVs, revealing their potential to modulate multiple human disease pathways. *In vitro* and *in vivo* experiments demonstrated that BB-DEVs can regulate the levels of key inflammatory cytokines, including IL-6, TNF-α, and IL-10. Furthermore, *in vivo* studies showed that BB-DEVs enhance the phosphorylation of PI3K, AKT, and mTOR proteins, thereby promoting burn wound healing in mice.

## 5 Conclusion

In summary, this study shows that BB-DEVs can be involved in the regulation of many human diseases. It can also promote the healing of burn wounds by exerting anti-inflammatory effects and activating the PI3K-Akt pathway.

## Data Availability

The datasets presented in this study can be found in online repositories. The names of the repository/repositories and accession number(s) can be found below: https://www.ncbi.nlm.nih.gov/, PRJNA1370818.
