# Supplementary material for: The effectiveness and potential regulatory mechanism of Blumea balsamifera derived extracellular vesicles in promoting burn wound healing
Source: Front Cell Dev Biol. 2026 Mar 19;14:1756718. doi: 10.3389/fcell.2026.1756718 (PMC13044083; doi:10.3389/fcell.2026.1756718)
Supplement: Supplementary file 1 [file DataSheet1.zip › Supplementary Materials/Table S1Components from BB-ELNs.docx]

| **NO.** | **Retention time**  **(min)** | **Compound** | **Relative content ( % )** | **CAS** | **Formula** |
| --- | --- | --- | --- | --- | --- |
| 1 | 9.249 | 2-Propenamide | 0.572 | 79-06-1 | C3H5NO |
| 2 | 9.271 | 2-Octanol | / | 123-96-6 | C8H18O |
| 3 | 9.275 | Cyanic acid, ethyl ester | 0.171 | 627-48-5 | C3H5NO |
| 4 | 12.136 | Linalool | 0.140 | 78-70-6 | C10H18O |
| 5 | 13.440 | (+)-2-Bornanone | 1.529 | 464-49-3 | C10H16O |
| 6 | 13.448 | Benzenamine, 4-ethoxy- | 0.424 | 156-43-4 | C8H11NO |
| 7 | 13.449 | 4(1H)-Pyridinone, 1,2,6-trimethyl- | 0.349 | 767-83-9 | C8H11NO |
| 8 | 14.171 | Butanoic acid, anhydride | 0.288 | 106-31-0 | C8H14O3 |
| 9 | 14.176 | 3-Butyn-2-one | 0.574 | 1423-60-5 | C4H4O |
| 10 | 14.194 | di-t-Butyl phosphite | 0.838 | 13086-84-5 | C8H19O3P |
| 11 | 14.213 | Ethanone, 1-cyclopropyl-2-(4-pyridinyl)- | 2.347 | 6580-95-6 | C10H11NO |
| 12 | 14.217 | 1H-Pyrrole-3-carboxylic acid | 0.931 | 931-03-3 | C5H5NO2 |
| 13 | 14.218 | 2-Pentyn-4-one | 1.646 | 7299-55-0 | C5H6O |
| 14 | 14.230 | 2-Furancarboxylic acid, anhydride | 21.353 | 615-08-7 | C10H6O5 |
| 15 | 14.230 | 2,5-pyrrolidinedione, 1-[(2-furanylcarbonyl)oxy]- | 21.336 | 1010400-58-7 | C9H7NO5 |
| 16 | 14.771 | .alpha.-Terpineol | 0.061 | 98-55-5 | C10H18O |
| 17 | 14.935 | 1,5-Cyclohexadiene-1-methanol, 4-(1-methylethyl)- | 0.032 | 19876-45-0 | C10H16O |
| 18 | 15.360 | Lilac alcohol B | 0.057 | 33081-35-5 | C10H18O2 |
| 19 | 15.534 | 6-Octen-1-ol, 7-methyl-3-methylene- | 0.098 | 13066-51-8 | C10H18O |
| 20 | 15.789 | 1-Cyclopentene-1-methanol, 2-methyl-5-(1-methylethyl)- | 0.046 | 80113-82-2 | C10H18O |
| 21 | 16.149 | Benzaldehyde, 4-(1-methylethyl)- | 0.073 | 122-03-2 | C10H12O |
| 22 | 16.433 | Thymoquinone | 0.086 | 490-91-5 | C10H12O2 |
| 23 | 17.592 | Thymol | 0.949 | 89-83-8 | C10H14O |
| 24 | 17.770 | (S)-(-)-(4-Isopropenyl-1-cyclohexenyl)methanol | 0.075 | 18457-55-1 | C10H16O |
| 25 | 18.488 | Silphiperfol-5-ene | 3.064 | 138752-24-6 | C15H24 |
| 26 | 19.000 | 7-epi-Silphiperfol-5-ene | 5.921 | 138752-23-5 | C15H24 |
| 27 | 19.000 | Demethoxyencecalinol | 0.937 | 71822-00-9 | C13H16O2 |
| 28 | 19.118 | 3a,7-Methano-3aH-cyclopentacyclooctene, 1,4,5,6,7,8,9,9a-octahydro-1,1,7-trimethyl-, [3aR-(3a.alpha.,7.alpha.,9a.beta.)]- | 0.038 | 469-92-1 | C15H24 |
| 29 | 19.327 | Silphiperfola-4,7(14)-diene | 0.202 | 210637-49-3 | C15H22 |
| 30 | 19.819 | 2-[p-Methoxybenzyloxy]-6-methoxy-8-nitroquinoline | 0.039 | 63624-50-0 | C18H16N2O5 |
| 31 | 19.904 | 2,3-Bornanediol | 0.073 | 25696-56-4 | C10H18O2 |
| 32 | 20.130 | (1R,3aS,5aS,8aR)-1,3a,4,5a-Tetramethyl-1,2,3,3a,5a,6,7,8-octahydrocyclopenta[c]pentalene | 0.051 | 65372-78-3 | C15H24 |
| 33 | 20.717 | isoledene | 0.075 | 95910-36-4 | C15H24 |
| 34 | 20.832 | 2,4,7,9-Tetramethyl-5-decyn-4,7-diol | 0.044 | 126-86-3 | C14H26O2 |
| 35 | 20.988 | 9H-Fluorene, 1,9-dimethyl- | 0.155 | 17057-98-6 | C15H14 |
| 36 | 20.999 | Bicyclo[5.2.0]nonane, 2-methylene-4,8,8-trimethyl-4-vinyl- | 5.739 | 242794-76-9 | C15H24 |
| 37 | 21.415 | 4-(1,2-Dimethyl-cyclopent-2-enyl)-butan-2-one | 0.059 | 75698-06-5 | C11H18O |
| 38 | 21.827 | 2,4-Cyclopentadiene-1-ethanamine | 0.126 | 138816-65-6 | C7H11N |
| 39 | 22.004 | Alloaromadendrene | 0.325 | 25246-27-9 | C15H24 |
| 40 | 22.346 | 4a,8-Dimethyl-2-(prop-1-en-2-yl)-1,2,3,4,4a,5,6,7-octahydronaphthalene | 0.046 | 103827-22-1 | C15H24 |
| 41 | 22.571 | 1-Benzoxepin, 2,3,4,5-tetrahydro- | 0.160 | 6169-78-4 | C10H12O |
| 42 | 22.773 | Phenol, 3-(1,1-dimethylethyl)-4-methoxy- | 0.821 | 88-32-4 | C11H16O2 |
| 43 | 22.946 | Benzenepropanoic acid, .beta.-hydroxy-, methyl ester | 0.030 | 7497-61-2 | C10H12O3 |
| 44 | 23.091 | 2-Heptanone, 6-methyl-6-[3-methyl-3-(1-methylethenyl)-1-cyclopropen-1-yl]- | 0.050 | 69296-87-3 | C15H24O |
| 45 | 23.217 | 2,4-Di-tert-butylphenol | 0.566 | 96-76-4 | C14H22O |
| 46 | 23.219 | (Benzo(b)thien-6-yl)acetic acid | 0.104 | 6177-87-3 | C10H8O2S |
| 47 | 23.441 | 1,4-Benzenediamine, N-(1-methylheptyl)- | 0.194 | 39563-50-3 | C14H24N2 |
| 48 | 23.540 | 1-Naphthalenemethanol, 1,4,4a,5,6,7,8,8a-octahydro-2,5,5,8a-tetramethyl- | 0.251 | 19078-37-6 | C15H26O |
| 49 | 23.994 | .alpha.-Calacorene | 0.025 | 21391-99-1 | C15H20 |
| 50 | 24.124 | 1H-3a,7-Methanoazulen-5-ol, octahydro-3,8,8-trimethyl-6-methylene- | 0.119 | 28231-03-0 | C15H24O |
| 51 | 24.265 | 6-Isopropenyl-4,8a-dimethyl-4a,5,6,7,8,8a-hexahydro-1H-naphthalen-2-one | 0.088 | 86917-79-5 | C15H22O |
| 52 | 24.424 | Bornyl angelate | 0.178 | 85758-37-8 | C15H24O2 |
| 53 | 24.617 | 4aH-Cycloprop[e]azulen-4a-ol, decahydro-1,1,4,7-tetramethyl-, [1aR-(1a.alpha.,4.beta.,4a.beta.,7.alpha.,7a.beta.,7b.alpha.)]- | 0.161 | 5986-49-2 | C15H26O |
| 54 | 24.801 | 1(2H)-Naphthalenone, 3,4,4a,5,6,7-hexahydro-4a,5-dimethyl-3-(1-methylethenyl)-, [3S-(3.alpha.,4a.alpha.,5.alpha.)]- | 0.270 | 562-23-2 | C15H22O |
| 55 | 24.988 | 1,3-Cyclopentadiene, 1,3-bis(1-methylethyl)- | 0.638 | 123278-27-3 | C11H18 |
| 56 | 24.990 | Caryophyllene oxide | 1.575 | 1139-30-6 | C15H24O |
| 57 | 25.194 | 1-Naphthalenol, decahydro-1,4a-dimethyl-7-(1-methylethylidene)-, [1R-(1.alpha.,4a.beta.,8a.alpha.)]- | 0.071 | 473-04-1 | C15H26O |
| 58 | 25.294 | D-Bicuculline | 0.092 | 485-49-4 | C20H17NO6 |
| 59 | 25.301 | Guaiol | 1.436 | 489-86-1 | C15H26O |
| 60 | 25.456 | 2-Ethyl-5-undecyl-5-pyrroline | 0.117 | 114640-30-1 | C17H33N |
| 61 | 25.456 | (Z)-2-(henicos-12-en-1-yl)-6-methyl-2H-pyran-4(3H)-one | 0.128 | 243118-19-6 | C27H48O2 |
| 62 | 25.457 | Ledol | 0.469 | 577-27-5 | C15H26O |
| 63 | 25.492 | .alpha.-epi-7-epi-5-Eudesmol | 0.330 | 446050-56-2 | C15H26O |
| 64 | 25.556 | Di(2-picolyl)amine | 0.117 | 1539-42-0 | C12H13N3 |
| 65 | 25.560 | 1-Methylbicyclo[2.2.1]heptan-exo-2-ol | 0.169 | 766-25-6 | C8H14O |
| 66 | 25.564 | 2-Naphthalenemethanol, 2,3,4,4a,5,6,7,8-octahydro-.alpha.,.alpha.,4a,8-tetramethyl-, [2R-(2.alpha.,4a.beta.,8.beta.)]- | 0.546 | 63891-61-2 | C15H26O |
| 67 | 25.684 | 2,10,10-Trimethyltricyclo[7.1.1.0(2,7)]undec-7-en-6-one | 0.107 | 148323-04-0 | C14H20O |
| 68 | 25.837 | 3-[4-(2-Methylpropyl)phenyl]butan-2-one | 0.337 | 64758-90-3 | C14H20O |
| 69 | 25.839 | 2-Naphthalenemethanol, 1,2,3,4,4a,5,6,7-octahydro-.alpha.,.alpha.,4a,8-tetramethyl-, (2R-cis)- | 1.192 | 1209-71-8 | C15H26O |
| 70 | 26.094 | 10-epi-.gamma.-Eudesmol | 0.919 | 15051-81-7 | C15H26O |
| 71 | 26.096 | Benzenecarbothioic acid, 2,4,6-triethyl-, S-(2-phenylethyl) ester | 0.214 | 64712-67-0 | C21H26OS |
| 72 | 26.128 | 11,11-Dimethyl-4,8-dimethylenebicyclo[7.2.0]undecan-3-ol | 0.699 | 79580-01-1 | C15H24O |
| 73 | 26.128 | Benzenemethanol, 4-methoxy-.alpha.-(2-nitrocyclopentyl)-, [1.alpha.(R*),2.alpha.]- | 0.324 | 103130-05-8 | C13H17NO4 |
| 74 | 26.215 | 10,10-Dimethyl-2,6-dimethylenebicyclo[7.2.0]undecan-5.beta.-ol | 1.783 | 19431-80-2 | C15H24O |
| 75 | 26.376 | 2H-1-Benzopyran, 2,2-dimethyl- | 0.146 | 2513-25-9 | C11H12O |
| 76 | 26.377 | N-(1H-Benzimidazol-1-yl)-2-phenylacetamide | 0.087 | 380388-72-7 | C15H13N3O |
| 77 | 26.449 | Benzo[b]thiophene, 2-ethyl- | 0.031 | 1196-81-2 | C10H10S |
| 78 | 26.614 | p-n-Butylacetophenone | 0.320 | 37920-25-5 | C12H16O |
| 79 | 26.621 | 1,2-Dihydro-3,6-diphenyl-S-tetrazine | 0.045 | 14478-73-0 | C14H12N4 |
| 80 | 26.625 | 5-(4-Methoxyphenyl)pentanoic acid | 0.165 | 7508-04-5 | C12H16O3 |
| 81 | 26.635 | Neointermedeol | 2.401 | 5945-72-2 | C15H26O |
| 82 | 27.004 | 2-((2R,4aR,8aR)-4a,8-Dimethyl-1,2,3,4,4a,5,6,8a-octahydronaphthalen-2-yl)prop-2-en-1-ol | 0.543 | 65018-15-7 | C15H24O |
| 83 | 27.009 | 1-(2-Acetyloxy-5-methylphenyl)propan-1-one | 0.125 | 392721-71-0 | C12H14O3 |
| 84 | 27.023 | 2-(6-Methoxypyridin-3-yl)ethanamine | 0.308 | 154403-89-1 | C8H12N2O |
| 85 | 27.028 | Pyrrole-2,5-dicarboxylic acid, N,O,O'-trimethyl | 0.920 | 1757-30-8 | C9H11NO4 |
| 86 | 27.032 | Xanthoxylin | 8.527 | 90-24-4 | C10H12O4 |
| 87 | 27.780 | Pentadecanal- | 0.139 | 2765-11-9 | C15H30O |
| 88 | 28.218 | 2H-Cycloprop[c]indene-2,3(3ah)-dione, hexahydro-3a,7,7-trimethyl- | 0.081 | 96678-99-8 | C13H18O2 |
| 89 | 29.071 | Cyperolactone | 0.117 | 1940169-14-1 | C15H22O2 |
| 90 | 30.353 | 7-(2-Hydroxypropan-2-yl)-1,4a-dimethyldecahydronaphthalen-1-ol | 0.686 | 92857-25-5 | C15H28O2 |
| 91 | 31.383 | Palmitoleic acid | 0.045 | 373-49-9 | C16H30O2 |
| 92 | 35.529 | Hexadecanoic acid, ethyl ester | 0.042 | 628-97-7 | C18H36O2 |
| 93 | 36.076 | 2-Penten-1-one, 1-(2-hydroxy-5-methylphenyl)-4-methyl- | 0.037 | 51956-80-0 | C13H16O2 |
| 94 | 38.351 | Cyclohexene, 6-ethenyl-6-methyl-1-(1-methylethyl)-3-(1-methylethylidene)-, (S)- | 0.051 | 5951-67-7 | C15H24 |
| 95 | 39.499 | 1-Cyclopropene-1-pentanol, .alpha.,.epsilon.,.epsilon.,2-tetramethyl-3-(1-methylethenyl)- | 0.026 | 90165-06-3 | C15H26O |
